# Golgi-Cox Staining Step by Step

**DOI:** 10.3389/fnana.2016.00038

**Published:** 2016-03-31

**Authors:** Sami Zaqout, Angela M. Kaindl

**Affiliations:** ^1^Institute of Cell Biology and Neurobiology, Charité – Universitätsmedizin BerlinBerlin, Germany; ^2^Center for Chronically Sick Children (Sozialpädiatrisches Zentrum, SPZ), Charité – Universitätsmedizin BerlinBerlin, Germany

**Keywords:** Golgi, neuronal morphology, spines, dendrites, vibratome

## Abstract

Golgi staining remains a key method to study neuronal morphology *in vivo*. Since most protocols delineating modifications of the original staining method lack details on critical steps, establishing this method in a laboratory can be time-consuming and frustrating. Here, we describe the Golgi-Cox staining in such detail that should turn the staining into an easily feasible method for all scientists working in the neuroscience field.

## Introduction

Studying neuronal morphology is more relevant than ever given the rapid identification of novel genetic neurodevelopmental diseases through next-generation sequencing approaches and the subsequent need to understand underlying pathomechanisms. Discovered already by Golgi (1873), the non-invasive Golgi staining method is far from out-of-date, and it facilitates an analysis of neuronal morphology with axonal and dendritic arborization and spines through visualization of only a low percentage of neurons (1–3%). Three major Golgi staining subtypes exist: Rapid Golgi, Golgi–Kopsch, and Golgi–Cox (Koyama, 2013). Of these, the Golgi-Cox method is considered to be most reliable in demonstrating dendritic arborization with a low background (Buell, 1982; Koyama, 2013). Many modifications of this method have been conducted and most aimed to increase its reliability (Angulo et al., 1996; Gibb and Kolb, 1998; Koyama and Tohyama, 2012), to reduce the required time (Ranjan and Mallick, 2010; Levine et al., 2013; Patro et al., 2013), and to increase the selectivity of neuronal vs. glial staining or* vice versa* (Ranjan and Mallick, 2012; Gull et al., 2015). Also, commercial kits have been developed for relatively fast constant Golgi staining. However, most Golgi staining descriptions lack exact details of individual steps. Here, we report in detail all steps of the Golgi-Cox staining method on adult mouse brain vibratome sections required for a reliable high quality staining in an acceptable time frame and with well-preserved tissue quality. The materials applied are available in most neuroscience labs and sufficient to establish the Golgi-Cox staining for many samples. Following our protocol step-by-step will likely minimize troubles encountered frequently and reduce the time required to standardize this method.

## Materials, Equipment and Stepwise Procedures

### Mice

Adult 6–12-week-old* C57BL/6* mice were obtained from the animal facility of the Charité—Universitätsmedizin Berlin, Germany. All experiments were carried out in accordance to the national ethic principles (registration no. T0309.09).

### General Instructions Before Starting

1. All glass and plastic bottles should be rinsed with fresh double distilled water (dd-H_2_O) before use.
2. Plastic-coated magnetic stirring pills (rods) can be used to dissolve all chemicals in dd-H_2_O properly.
3. Metal instruments must be avoided during the impregnation step.
4. The amount of solutions and gelatin-coated slides should be prepared according to the number of samples as indicated in each section.
5. The incubation time frame given for various steps in this protocol are periods that do not affect significantly the staining quality. Thus, these time frames provide some flexibility when pursuing with the protocol.
6. All solutions must be stored in the dark using aluminum foil to cover large bottles, or by placing small bottles in a covered, lightproof box.
7. Care should be taken with all solutions due to toxicity and carcinogenesis: direct skin contact and inhalation can be avoided by wearing gloves and performing the experiments under a chemical hood, respectively.

### Preparation of Solutions

#### Solution for Sample Impregnation

The impregnation stock solutions are prepared by dissolving 15 g from of the following chemicals in 300 ml dd-H_2_O (5% w/v) each:

1. Potassium dichromate (K_2_ Cr_2_ O_7_; 1.04862, Merck KGaA, Germany).
2. Mercuric chloride (HgCl_2_; KK04.2, Carl Roth GmbH, Germany).
3. Potassium chromate (K_2_CrO_4_; HN33.2, Carl Roth GmbH, Germany).

All three solutions, stored in bottles at room temperature in the dark, are for long-term usage to prepare Golgi-Cox solution. These solutions are sufficient for 36 adult mouse brains. Golgi-Cox solution is prepared for each experiment in a new bottle using the three stock solutions mentioned above as follows (Rutledge et al., 1969).

1. 50 ml of the potassium dichromate solution is mixed with 50 ml of the mercuric chloride solution.
2. 40 ml of the potassium chromate solution is added.
3. 100 ml of dd-H_2_O is added.

After mixing the solution, the bottle needs to be covered with aluminum foil and kept to settle at room temperature for at least 48 h before use to allow precipitate formation. This solution is sufficient for six adult mouse brains and can be used for up to 1 month.

#### Solution for Tissue Protection

First, 0.1 M phosphate buffer (pH 7.2) is prepared by dissolving the following components together in 500 ml dd-H_2_O:

1. 1.59 g sodium-hydrogen-phopsphate monohydrate (NaH_2_P0_4_ ·H_2_0; T878.3, Carl Roth GmbH, Germany).
2. 5.47 g di-sodium-hydrogen-phosphate water-free (Na_2_HP0_4_; P030.2, Carl Roth GmbH, Germany).
3. 9.0 g sodium chloride (NaCl; 9265.1, Carl Roth GmbH, Germany).

Second, 1000 ml of a tissue-protectant (cryoprotectant) solution (de Olmos et al., 1978; Watson et al., 1986) is prepared by dissolving the following components in the previous 500 ml phosphate buffer:

1. 300 g sucrose (C_12_H_22_O_11_; 1.07687, Merck KGaA, Germany).
2. 10 g polyvinylpyrrolidone (PVP40, Sigma-Aldrich, Germany).
3. 300 ml ethylene glycol (C_2_H_6_O_2_; E-9129, Sigma-Aldrich, Germany).

The final volume is then adjusted to 1000 ml with dd-H_2_O. 500 ml of the solution can be kept in a separate bottle to fill the vibratome chamber and the rest is sufficient to pursue with the tissue protection step for 25 adult mouse brains. This solution needs to be stored at 4°C in the dark for long term storage.

#### Solutions for the Developing Step

For the developing step, about 300 ml from each of the following solutions are needed:

1. 50, 70, 95, and 100% ethanol series (C_2_H_6_O; K928.4, Carl Roth GmbH, Germany).
2. Xylene (C_8_H_10_; 9713.3, Carl Roth GmbH, Germany).
3. 3:1 ammonia:dd-H_2_O is prepared by mixing 200 ml ammonia (NH_3_.H_2_O; 6774.2, Carl Roth GmbH, Germany) with 100 ml dd-H_2_O.
4. 5% sodium thiosulfate is prepared by dissolving 15 g sodium thiosulfate (Na_2_S_2_O_3_.5H_2_O; 2781895, Merck KGaA, Germany) in 300 ml dd-H_2_O.

All solutions are stored at room temperature and the bottle with the sodium thiosulfate solution is covered with aluminum foil. These solutions can be re-used and should be replaced when they turn dark.

#### Solutions and Materials for Gelatin-Coated Slides

Seventy five plain microscopic slides (micro slides; 2406/1, Glaswarenfabrik Karl Hecht GmbH and Co KG) are first placed in three staining racks (2285.1, Carl Roth GmbH, Germany), washed thoroughly with dd-H_2_O and kept for drying in a dust-free area (e.g., under a chemical hood) for 2–3 h. In the meantime, 3% gelatin is prepared by dissolving 9 g gelatin from porcine skin (Type A; G2500, Sigma-Aldrich, Germany) in 300 ml dd-H2O with constant stirring and heating to 55°C. The solution is then filtered with filter paper (240 mm; 4.303.240, Neolab, Germany) into a clean histological staining box (2285.1, Carl Roth GmbH, Germany). A rack with the cleaned slides is immersed into the warm gelatin solution for 10 min, subsequently placed on plenty tissue papers and kept at room temperature in a dust-free area overnight. The gelatin should be re-warmed to 55°C before immersing a further rack of slides. These slides are sufficient for 200 μm thick-brain sections collected from six adult mouse brains. If more than three racks are needed, more 3% gelatin solution needs to be prepared. These gelatin-coated slides need to be stored in closed histological staining boxes and are best to be used within a month of preparation.

### Impregnation Step

For each brain sample, one small bottle (multi-purpose container with lid; 203170, Greiner bio-one, Germany) is washed with dd-H_2_O. The aluminum foils are removed gently from the Golgi-Cox bottle without shaking, to avoid the solution mixing with brownish precipitates at the bottom of the bottle. For each sample, 10 ml is taken from the upper clear part of Golgi-Cox solution and dispensed into each small bottle.

**Figure 1:**
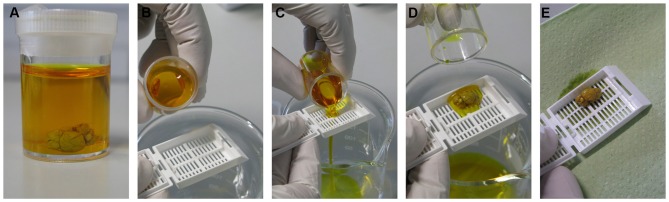
**Impregnation step.** The brain sample is kept in Golgi-Cox solution at room temperature in the dark **(A)**. After 24 h, the sample is transferred into a new Golgi solution-containing bottle with the help of a histological cassette as shown in the serial pictures **(B–E)** and kept to settle at room temperature in dark for 7–10 days.

After cervical dislocation, the brain is dissected quickly but carefully, washed with dd-H_2_O, and cut into two halves to allow better impregnation. Each half is then transferred into an individual small bottle with Golgi-Cox solution (Figure 1A) and stored at room temperature in dark. Fixation or perfusion with 4% PFA of adult brains should be avoided because this leads to over-impregnated neurons rendering a study of neuronal arborization impossible. After 24 h, each brain sample is transferred into a new small bottle of Golgi-Cox solution using plastic forceps or preferably by pouring the solution and sample into histological cassettes (Rotilabo^®^ embedding cassettes; K114.1, Carl Roth GmbH, Germany) as shown in Figures 1B–E. The small bottles are kept at room temperature in the dark for 7–10 days.

### Tissue Protection Step

**Figure 2:**
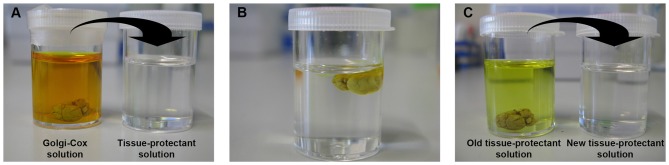
**Tissue protection step.** The brain sample is transferred from the Golgi-Cox impregnation solution to a new bottle with tissue-protectant solution and kept at 4°C in the dark **(A,B)**. After 24 h, the tissue-protectant solution is replaced by a fresh solution in a new bottle **(C)**.

For each brain sample, one small bottle, as described above, is washed with dd-H_2_O and filled with 10 ml tissue-protectant solution. With the help of histological cassettes, as described above, each brain sample is transferred from Golgi-Cox solution to tissue-protectant solution (Figures 2A,B), and stored at 4°C in dark. After 24 h, each brain sample is transferred into a new small bottle with 10 ml tissue-protectant solution (Figure 2C). The small bottles are kept at 4°C in dark for 4–7 days.

### Sectioning Step

**Figure 3:**
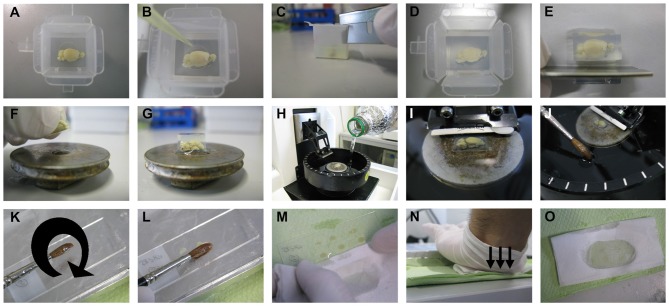
**Sectioning step.** The brain sample is embedded in 4% low melting point agarose. After pouring the agarose solution on the brain and thus into the embedding mold, the position of the brain needs to be adjusted by keeping the cut side to the bottom of the mold using a pipette tip **(A,B)**. After complete hardening of the agarose, the mold is cut at the edges, then at the sides, and the surplus agarose around the sample is further trimmed with a razor blade **(C–E)**. The trimmed agarose block is fixed to a vibratome plate **(F,G)**, and the plate is placed into the vibratome chamber, which is subsequently filled with tissue-protectant solution just until the agarose block is well covered **(H,I)**. Using a thick brush, sections are collected from the vibratome chamber and transferred onto gelatin-coated slides **(J–L)**. The surplus tissue-protectant solution around the sections is cleaned off with tissue paper, and the sections are blotted onto the slides by applying direct, downward moderate pressure with the heel of the palm **(M–O)**.

For our procedure, vibratome (Microm; HM_650V, Thermo Fisher Scientific Inc., Germany) was used for tissue sectioning as described below and shown in Figure 3. Cryostat or sliding-freezing microtome should also work fine as fully described in the protocol of the commercially available FD Rapid GolgiStain^™^ Kit (FD Neurotechnologies, Inc., MD, USA). For sectioning using a vibratome, 4% agarose is prepared by dissolving 2 g low melting point agarose (V2111, Promegain, WI, USA) in 50 ml dd-H2O by stirring first and then using a microwave until completely dissolved. Brain samples are then gently dried on tissue paper with the help of histological cassettes, as described above, and transferred into disposable plastic embedding molds (18646A, Polysciences Inc., PA, USA; Figure 3A). When the agarose temperature has cooled down to 47°C, it is poured into the molds until the brain samples are well covered with solution. The orientation of the brain needs to be adjusted with a pipette tip by keeping the medial cut side of the brain at the bottom of the mold (Figure 3B). If the most medial part of the brain sample is needed for sectioning, a thin layer of agarose should be poured into the bottom of the mold, and the mold should then be kept at 4°C for hardening. The brain sample can be then transferred as described above. After complete hardening of agarose, the edges of the molds are cut using a razor blade and surplus agarose around the brain sample is trimmed (Figures 3C–E).

With one drop of fast glue (Instant adhesive; E10C589, Best-CA, Germany), the brain sample is fixed to the vibratome plate (Figures 3F,G), and the plate is placed in the vibratome chamber. Subsequently, the vibratome chamber is filled with tissue-protectant solution and the cutting razor blade (Sward Classic, 7000115z, Wilkinson GmbH, Germany) put in place (Figures 3H,I). In our hands, a vibration frequency of 60 Hz and a speed to 15 mm/s works best, but these values can be changed according to the quality of cutting. The section thickness is optimally 200 μm for dendritic arborization studies and 100 μm for dendritic spines studies. While cutting, sections are collected with a thick brush and transferred onto gelatin-coated slides (Figures 3J–L). As soon as all sections have been loaded on slides, the surplus tissue-protectant solution around the sections is cleaned off with absorbent paper (Figure 3M). The sections are then blotted by pressing an absorbent paper moistened with tissue-protectant solution onto the slides. The best way, as described previously, is by applying direct, downward moderate pressure with the heel of the palm (Gibb and Kolb, 1998; Figures 3N,O). If the sections stick to the absorbent paper, then the paper is likely not soaked sufficiently with tissue-protectant solution. The slides with sections are transferred to racks and kept for drying in dark for 2–3 days.

After finishing the sectioning of all samples, the tissue-protectant solution in the vibratome chamber can be filtered with filter paper (240 mm; 4.303.240, Neolab, Germany) into a bottle, kept at 4°C in dark and re-used several times. However, this filtered tissue-protectant solution should be used only to fill the vibratome chamber and not for the tissue protection step.

### Developing Step

**Figure 4:**
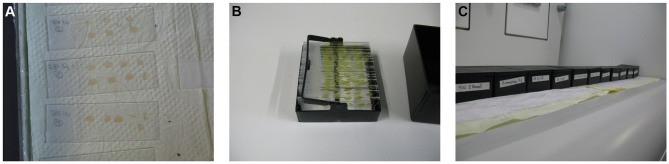
**Developing step.** After drying the sections, they are transferred to staining racks **(A,B)** and developed as described in the manuscript text **(C)**.

Using common histological staining boxes, the racks with slides are dehydrated and developed as follows (Figure 4):

1. Distilled water twice for 5 min each.
2. 50% ethanol for 5 min.
3. 3:1 ammonia solution for 8 min.
4. Distilled water twice for 5 min each.
5. 5% sodium thiosulfate for 10 min in dark.
6. Distilled water twice for 1 min each.
7. Optionally, sections can be incubated in 1% cresyl violet (as a counterstain) for 5 min.
8. 70, 95, and 100% ethanol for 6 min each.
9. Xylol for 6 min, and the sections can be kept in xylol longer until the mounting step.

We found that these steps are sufficient to receive good results; however the last two steps (dehydration with ethanol and cleaning with xylol) can be duplicated for further quality improvement.

### Mounting Step

For mounting, only two slides are taken from the xylol box per step and kept for about 1 min until they are semidry. Depending on the thickness of the sections, 5 (for 100 μm thick-sections) to 10 (for 200 μm thick-sections) drops of Eukitt (quick-hardening mounting medium; 03989, Fluka Analytical, Germany) are added. The slides are then covered with cover glass and air bubbles avoided by applying light pressure. After finishing the mounting of all samples, the slides are sealed with nail polish. The slides are then kept in a horizontal position for draying in the dark for 48 h before imaging. The sections can be subsequently stored in slide boxes in the dark at room temperature for long time usage.

### Imaging

In our laboratory, 1-μm-spaced Z-stack brightfield images for dendritic arborization studies are optimal in our hands and are taken by an Olympus IX81 microscope equipped with a F View II (sw) camera (Soft Imaging System GmbH, Münster, Germany). Brightfield images for dendritic spines are taken by Olympus BX60 microscope with Axiocam MRc Zeiss camera and Axiovision 4.8 Software (Zeiss, Göttingen, Germany). All images are processed using Adobe Photoshop CS6 version 13.0 × 64 and ImageJ Software. The magnification and quality of the spine images can be increased using Transform J Scale plug-in available for the ImageJ Software.

## Results and Discussion

**Figure 5:**
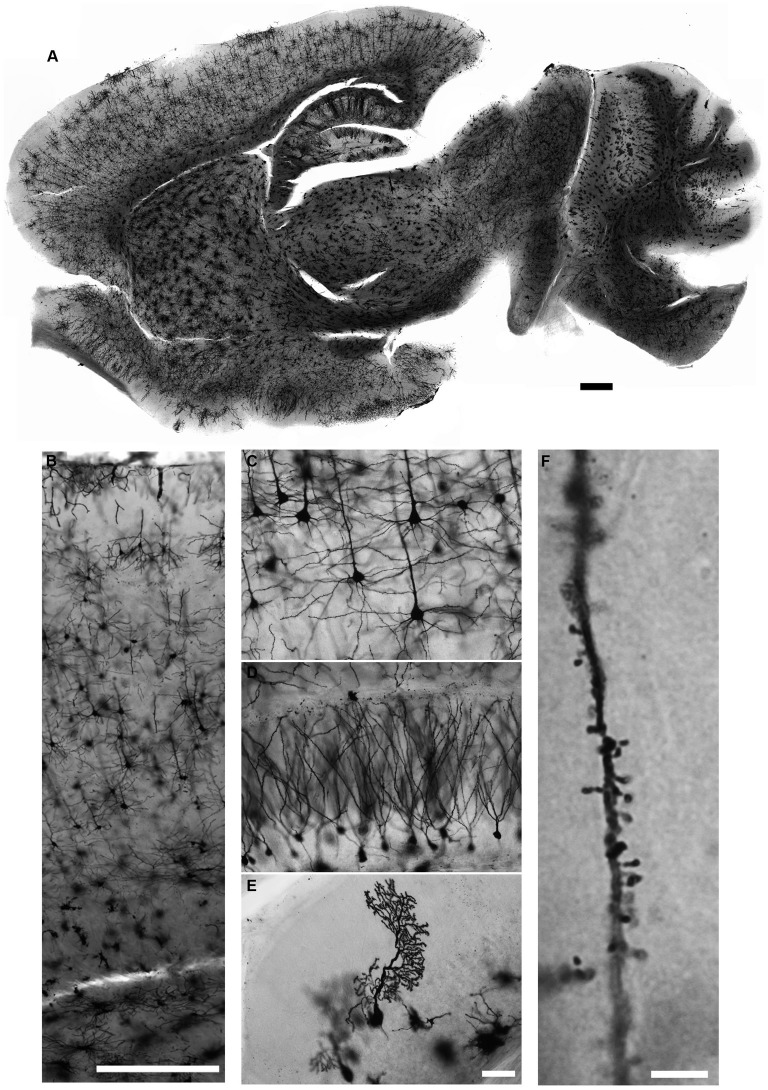
**Golgi-Cox staining for adult mouse brain.** Neurons in all brain regions are evenly and reliably stained with the Golgi-Cox protocol described here **(A)**. Magnified images of cerebral cortex **(B,C)**, hippocampus **(D)** and cerebellar cortex **(E)**. Dendritic spines can also be visualized in much higher magnification **(F)**. (Golgi staining, DIC images, scale bars 500 μm in **(A,B)**, 50 μm in **(E)** and 5 μm in **(F)**).

**Figure 6:**
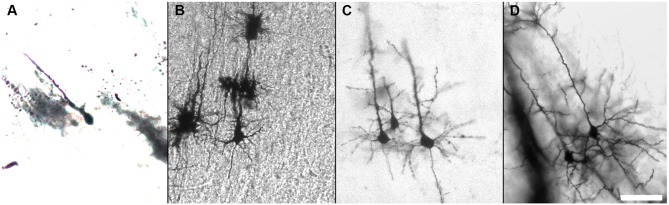
**Golgi-Cox staining of the developing mouse brain.** Impregnation of neurons of the developing mouse brain can be improved after brief fixation with 4% PFA as shown for P0 **(A)**, P5 **(B)**, P7 **(C)**, and P10 **(D)**. (Golgi staining, DIC images, scale bar 50 μm).

We describe in detail the Golgi-Cox staining protocol from preparation of the solutions, via transferring brain tissue, tissue sectioning, and development to mounting of the stained sections. Using this protocol, we have found that the dendritic tree and the dendritic spines of neurons are evenly and constantly stained in all brain regions (Figure 5). Additionally, while most Golgi-based studies report using coronal sections, we have found that neuronal dendritic arborization is best preserved and imaged when brains are cut in the sagittal plane, as also note by Valverde (1998). We also found that the tissue-protectant (cryoprotectant) solution (de Olmos et al., 1978; Watson et al., 1986) is very helpful to preserve tissue quality, reduce the staining background, and improve the attachment of sections to gelatin-coated slides. Minor additional steps can be added to our protocol to amend it for younger mice (Koyama and Tohyama, 2012) or to increase the staining for glia cells more than neurons (Ranjan and Mallick, 2012; Gull et al., 2015). Using our protocol, we found that initial brief fixation of brain samples with 4% PFA for 1 h followed by dd-H_2_O washing improves the staining for younger mice (Figure 6). Once all chemical and materials available in the lab, Golgi staining can be performed for large number of brain samples that lowers the overall cost needed to perform this staining.

## Conclusion

Applying this protocol renders Golgi staining easily feasible for all laboratories working on neuroscience projects.

## Author Contributions

SZ performed the experiments. SZ and AMK were responsible for the project conception and wrote as well as approved the final manuscript.

## Conflict of Interest Statement

The authors declare that the research was conducted in the absence of any commercial or financial relationships that could be construed as a potential conflict of interest.
